# A207 INFLIXIMAB IN VERY EARLY ONSET INFLAMMATORY BOWEL DISEASE (VEO-IBD), DOSING AND OUTCOME: A RETROSPECTIVE CROSS-SECTIONAL STUDY

**DOI:** 10.1093/jcag/gwae059.207

**Published:** 2025-02-10

**Authors:** A Sethuraman, S B Kishore, H Binomar, K Jacobson

**Affiliations:** Pediatric Gastroenterology, BC Children’s Hospital, Vancouver, BC, Canada; Gastroenterology, BC Children’s Hospital, Vancouver, BC, Canada; King Faisal Specialist Hospital and Research Centre, Riyadh, Riyadh, Saudi Arabia; Pediatric Gastroenterology, BC Children’s Hospital, Vancouver, BC, Canada

## Abstract

**Background:**

VEO-IBD patients defined as children with IBD diagnosed before 6rys of age, account for approximately 10% of pediatric IBD patients. They are uniquely at risk of inadequate infliximab (IFX) exposure. We report the dosing patterns, efficacy and safety of IFX use in a cohort of patients with VEO-IBD.

**Aims:**

To study the response rate and outcome of IFX in VEOIBD patients with active therapeutic drug monitoring. Our primary aim was to study the rate of clinical remission and outcome of patients with VEOIBD on IFX. Our secondary aim was to study the IFX failure rate in VEOIBD children and also to study the association of body weight (BW) and body surface area (BSA) dosing.

**Methods:**

A retrospective chart review was conducted for all children with VEOIBD diagnosed between 1^st^ January 2013 to 31^st^ December 2022 with a minimum follow up of one year at a single centre. The patients were identified from the British Columbia paediatric IBD database. Clinical data, including IBD medication use and response were collected from the medical record. REB approval was obtained for this study.

**Results:**

A total of 77 patients were identified and reviewed in our study with 28 (34.1%) exposed to infliximab, with a mean follow up of 65.6 months (±31.7mo). The mean age at diagnosis was 45.9 months (SD±18.5m). Among them, 14 (50%) were diagnosed with Crohn’s disease and 11 (39%) and 3 (11%) with ulcerative colitis and IBD-U respectively. 19/28 (67.8%) patients were started on IFX below 6yrs of age. The median time from diagnosis to IFX commencement was 11.9 months (IQR 3.9-11.9mo). The median pre dose 3 and pre dose 4 infliximab trough levels were 4.35 μg/mL and 5.2 μg /mL respectively with a mean IFX induction dose of 6.7 mg/kg (SD ±2.14mg/kg) and 160.6 mg/m^2^ body surface area. 18/28 (64.3%) patients needed a dose change after induction with the rest needing dose optimization at follow up. 12/28 (42.8%) were on concomitant therapy with methotrexate or azathioprine. 17/28 (60.7%) continued on IFX as their therapy, with a mean IFX dose of 300mg/m^2^ and 10.25 mg/kg to achieve clinical remission at last follow. The median drug level for these patients was 7.15 μg/mL.

Clinical remission at last follow up on IFX was achieved in all 17/28 patients with 7/17 in biochemical remission, defined at fecal calprotectin <250μg/g. Infliximab failure occurred in 11/28 (39.3%) of patients with 10/11 due to secondary loss of response. Among those 11, 7 were exposed to a 2^nd^ biologic and 4 to a 3^rd^ biologic with response in 7. Three patients required a colectomy and 1 underwent a bone marrow transplant.

**Conclusions:**

Infliximab trough levels pre dose 3 and 4 are often low in VEO-IBD patients and dose optimization is very common.

**Funding Agencies:**

None

